# Blocking representation in the ERA-Interim driven EURO-CORDEX RCMs

**DOI:** 10.1007/s00382-018-4335-8

**Published:** 2018-07-30

**Authors:** Martin Wolfgang Jury, Sixto Herrera, José Manuel Gutiérrez, David Barriopedro

**Affiliations:** 1 Wegener Center for Climate and Global Change, University of Graz, Brandhofgasse 5, 8010 Graz, Austria; 2 Meteorology Group, Department of Applied Mathematics and Computer Sciences, Universidad de Cantabria, 39005 Santander, Spain; 3 Meteorology Group, Institute of Physics of Cantabria, (CSIC-)Universidad de Cantabria, 39005 Santander, Spain; 4 Departamento de Física de la Tierra y Astrofísica, Universidad Complutense de Madrid (UCM), 28040 Madrid, Spain; 5 Instituto de Geociencias (IGEO), CSIC-UCM, 28040 Madrid, Spain

**Keywords:** Atmospheric blocking, Regional climate models, Temperature bias, Precipitation bias, Reanalysis driven, EURO-CORDEX

## Abstract

While Regional Climate Models (RCMs) have been shown to yield improved simulations compared to General Circulation Model (GCM), their representation of large-scale phenomena like atmospheric blocking has been hardly addressed. Here, we evaluate the ability of RCMs to simulate blocking situations present in their reanalysis driving data and analyse the associated impacts on anomalies and biases of European 2-m air temperature (TAS) and precipitation rate (PR). Five RCM runs stem from the EURO-CORDEX ensemble while three RCMs are WRF models with different nudging realizations, all of them driven by ERA-Interim for the period 1981–2010. The detected blocking systems are allocated to three sectors of the Euro-Atlantic region, allowing for a characterization of distinctive blocking-related TAS and PR anomalies. Our results indicate some misrepresentation of atmospheric blocking over the EURO-CORDEX domain, as compared to the driving reanalysis. Most of the RCMs showed fewer blocks than the driving data, while the blocking misdetection was negligible for RCMs strongly conditioned to the driving data. A higher resolution of the RCMs did not improve the representation of atmospheric blocking. However, all RCMs are able to reproduce the basic anomaly structure of TAS and PR connected to blocking. Moreover, the associated anomalies do not change substantially after correcting for the misrepresentation of blocking in RCMs. The overall model bias is mainly determined by pattern biases in the representations of surface parameters during non-blocking situations. Biases in blocking detections tend to have a secondary influence in the overall bias due to compensatory effects of missed blockings and non-blockings. However, they can lead to measurable effects in the presence of a strong blocking underestimation.

**Electronic supplementary material:**

The online version of this article (10.1007/s00382-018-4335-8) contains supplementary material, which is available to authorized users.

## Introduction

Regional Climate Models (RCMs) are a common tool to generate relevant climate information on regional scales (e.g. Dickinson et al. 1989; Giorgi and Bates 1989; Giorgi and Mearns 1991; Laprise 2008; Rummukainen 2010). Although, the choice of the driving General Circulation Model (GCM) is crucial in determining the overall uncertainty and the regional modeled fields (Déqué et al. 2007; Christensen and Christensen 2007), numerous studies have shown the improved representation of regional to local climate in RCMs due to their finer resolution and improved model physics and parameterizations (Prein et al. 2013; Ban et al. 2014; Prein et al. 2015; Torma et al. 2015; Giorgi et al. 2016). In recent years, there have been intensified efforts to identify regional changes with the help of RCMs over Europe (e.g. van der Linden and Mitchell 2009; Jacob et al. 2014).

Along with the added value of dynamical downscaling, there are possible downsides as well. For instance, there is the possibility that the RCM’s mean flow on the synoptic scale diverges from that of the GCM, especially if the regional domain is large enough (Jones et al. 1995; Diaconescu and Laprise 2013). This may hold benefits, since a better representation of certain phenomena might overcome some aspects of the “garbage in, garbage out” problem (Diaconescu and Laprise 2013; Hall 2014). On the contrary, different spectral or grid nudging techniques aim at conditioning a RCM more to its driving data, thus suppressing possible deviations from the larger scales (Kida et al. 1991; Storch et al. 2000; Rockel et al. 2008; Alexandru et al. 2009). One aspect hardly addressed in newer large downscaling experiments, like the Coordinated Regional Climate Downscaling Experiment (CORDEX, Giorgi et al. 2009; Evans 2011), is if the downscaling domain is large enough for RCMs to diverge from their driving GCMs, and, if so, whether RCMs better represent certain atmospheric phenomena or should be more strongly conditioned to their driving data.

Among these large-scale systems, blocking describes a situation where the westerly flow in the mid-latitudes is interrupted or deflected during several days to weeks by an anticyclonic high pressure system (Rex 1950). Due to its strong impact on European weather and climate, blocking has been thoroughly investigated in recent decades. Not only does blocking exert a strong influence on winter temperature extremes (Sillmann et al. 2011; Buehler et al. 2011; Sousa et al. 2017b), also major heatwaves over Europe were connected to blocking, as for instance the Russian heatwave 2010 (Matsueda 2011; Dole et al. 2011; Barriopedro et al. 2011; Schneidereit et al. 2012). The role of blocking in spring temperature extremes that mark the beginning of the European summer have also been discussed (Cassou and Cattiaux 2016; Brunner et al. 2017). Additionally, precipitation regimes are altered by blocking. Increased precipitation can be observed south and at the flanks of the blocked regions, while less precipitation occurs at the location of the blocking high (Buehler et al. 2011; Sousa et al. 2016, 2017a). Although the spread in blocking representation among the current generation of GCMs is high, overall GCMs tend to under-report blocking, especially in winter and over Europe (Masato et al. 2013a; Ja et al. 2013; Davini and D’Andrea 2016). Among other factors, a higher spatial resolution has often been shown to reduce this bias (Scaife et al. 2010; Dawson et al. 2012; Ja et al. 2013). This is thought to be related with a better representation of synoptic transient eddies, which act to maintain the block against dissipation through interactions with the large-scale flow (e.g. Shutts 1983; Yamazaki and Itoh 2009). In addition, Pithan et al. (2016) have suggested that blocking can also be improved by refining parameterizations, such as the low-level wave drag. Some of these crucial aspects as well as the local responses to blocking are arguably better resolved by RCMs (Whan et al. 2016).

Despite these overall advances in blocking representation in GCMs, the question persists whether RCMs are better able to reproduce blocking due to their higher resolution. In this paper we compare EURO-CORDEX RCMs with their reanalysis driving data in order to assess differences in blocking characteristics, including their associated impacts on surface anomalies. We further explore the contribution of blocking errors in RCMs to the climatological biases in surface variables, namely 2-m air temperature (TAS) and precipitation rate (PR).

## Data and methods

Several datasets covering the target period (1981–2010) have been used to define blocking events [based on geopotential height at 500 hPa (Z500)] and to analyse the effect of these events on the surface variables (TAS and PR) over the EURO-CORDEX domain (technical description on http://www.cordex.org/domains).

### Reanalyses and observations

The European Centre for Medium-Range Weather Forecasts (ECMWF) Interim reanalysis (ERA-Interim, Dee et al. 2011) has been considered as reference to define the blocking events, since it provided the lateral boundary conditions in the EURO-CORDEX evaluation experiments to drive the RCMs. To account for uncertainties in the ERA-Interim blocking diagnosis we also used daily-mean data of Z500 from two additional reanalysis products at different spatial resolutions (see Table 1): the Japanese 55-year reanalysis (JRA55, Kobayashi et al. 2015; Harada et al. 2016) and the 40-year National Centers for Environmental Prediction / National Center for Atmospheric Research reanalyses (NCEP/NCAR, Kalnay et al. 1996).

**Table 1:** Overview of the used reanalysis products

Name	Institution	Country	Horiz. Res.	References
ERA-Interim	European Centre for Medium-Range Weather Forecasts	Europe	0.75$$^\circ$$$$\times$$ 0.75$$^\circ$$	Dee et al. (2011)
JRA55	Japan Meteorological Agency	Japan	1.25$$^\circ$$$$\times$$ 1.25$$^\circ$$	Kobayashi et al. (2015); Harada et al. (2016)
NCEP/NCAR	National Centers for Environmental Prediction / National Center for Atmospheric Research reanalyses	USA	2.5$$^\circ$$$$\times$$ 2.5$$^\circ$$	Kalnay et al. (1996)

Columns denote the name, institution and country, horizontal resolution of the diagnostic grid and respective references of the single datasets

To validate and evaluate the surface fields in the RCMs, stations included in the European Climate Assessment & Dataset (ECA&D) and used in the COST Action Validating and Integrating Downscaling Methods for Climate Change Research (VALUE ECA 86 v2 dataset) have been considered (Maraun et al. 2015). This dataset contains daily precipitation and 2-m air temperature from 86 stations belonging to the blended dataset from the ECA&D Project (Klein Tank et al. 2002). The stations do not have more than 5% of missing values in the analysis period, and have been selected to cover the different European climates and regions with an homogeneous density.

### Regional climate models

The evaluation experiments of two different sets of RCM simulations have been considered in this study. First, daily Z500, TAS and PR data from three state-of-the-art RCMs of the EURO-CORDEX initiative (Jacob et al. 2014) at the horizontal resolutions of 0.44$^\circ$ have been used, namely, CCLM4-8-17, RACMO22E and RCA4. The two latter RCMs were additionally available at higher resolution (see Table 2 for an overview), which allowed to explore the effect of the horizontal resolution on the capability of the RCMs to reproduce blocking situations and their surface effects. Additionally, we extracted the same daily data from different configurations of the Weather Research and Forecasting (WRF) model (Skamarock et al. 2008), including the WRF configuration used in the EURO-CORDEX contribution of the Universidad de Cantabria (WRF-C) and two nudging approaches, spectral (WRF-SN) and grid (WRF-GN). All WRF models used the Grell-Devenyi cumulus parameterization (Grell and Dvnyi 2002), WRF single-moment (WSM-6) microphysics parameterization (similar to Hong and Chen 2004 with 6 species -vapor, cloud water, cloud ice, rain, snow and graupel- treated independently), the Noah land-surface model (Chen and Dudhia 2001), the Yonsei University planetary boundary layer (YSU PBL) diffusion package (Hong and Dudhia 2006), and the Community Atmosphere Model (CAM) radiation scheme (Collins et al. 2004). For both WRF nudging realizations, the respective (spectral or grid) technique was applied to the meridional and zonal wind, and to the geopotential, above the 10th level ($\sim$ 850 hPa), increasing linearly for the next upper five levels until about 600 hPa. While for spectral nudging (WRF-SN) the smallest wavelengths nudged were $\sim$11$^\circ$ ($\sim$ 1100–1200 km), grid nudging (WRF-GN) was applied equally to all wavelengths, without filtering the short-wave variability. These three WRF realizations enabled us to analyse if different nesting approaches, strongly linking the synoptic variables of the RCM with those of the reanalysis, improve the capability of the RCMs to reproduce blocking and associated impacts.

**Table 2:** Overview of the evaluated RCMs

Name	Institution	Country	Horiz. Res.	References
WRF-C	Universidad de Cantabria (UCAN)	Spain	0.44$$^\circ$$$$\times$$ 0.44$$^\circ$$	Menendez et al. (2014) and García-Díez et al. (2015)
WRF-SN				
WRF-GN				
CCLM4-8-17_44	Climate Limited-area Modelling Community (CLM-Community)	Europe	0.44$$^\circ$$$$\times$$ 0.44$$^\circ$$	Oleson et al. (2010)
RACMO22E_44	Royal Netherlands Meteorological Institute (KNMI)	Netherlands	0.44$$^\circ$$$$\times$$ 0.44$$^\circ$$	Meijgaard et al. (2008)
RACMO22E_11			0.11$$^\circ$$$$\times$$ 0.11$$^\circ$$	
RCA4_44	Swedish Meteorological and Hydrological Institute (SMHI), Rossby Centre	Sweden	0.44$$^\circ$$$$\times$$ 0.44$$^\circ$$	Samuelsson et al. (2011)
RCA4_11			0.11$$^\circ$$$$\times$$ 0.11$$^\circ$$	

Columns denote the name, institution and country, horizontal resolution and respective references of the single models

### Blocking detection

A multitude of detection methods to identify atmospheric blocking situations with gridded data exist in the literature, using either geopotential height or dynamic atmospheric fields like potential vorticity (e.g. Tibaldi and Molteni 1990; Pelly et al. 2003; Barriopedro et al. 2010; Scaife et al. 2010; Davini et al. 2012; Masato et al. 2013b). Here we apply a blocking index based on meridional differences of Z500 over a 2.5$$^\circ$$ latitude by 2.5$$^\circ$$ longitude grid, which localizes blocking high pressure systems between 55$$^\circ$$N and 65$$^\circ$$N (Barriopedro et al. 2006). Z500 data from reanalyses and RCMs have been bilinearily regridded to 2.5$$^\circ$$$$\times$$ 2.5$$^\circ$$. A blocking is detected if the criteria in Eqs. (1)–(3) are fulfilled for at least one of the five $$\varDelta$$ values and for five consecutive longitudes (12.5$$^\circ$$) over a period of at least five consecutive days:

1 $$\begin{aligned}&\dfrac{Z(\lambda ,\varPhi _0)-Z(\lambda ,\varPhi _S)}{\varPhi _0 - \varPhi _S} \ge 0 , \end{aligned}$$

2 $$\begin{aligned}&{\dfrac{Z(\lambda ,\varPhi _N)-Z(\lambda ,\varPhi _0)}{\varPhi _N - \varPhi _0} \le -10 \,\,{\text {m}}/{\text{deg}}} , \end{aligned}$$

3 $$\begin{aligned}&Z(\lambda ,\varPhi _0)-\overline{Z(\lambda ,\varPhi _0)}> 0 , \end{aligned}$$

$$\begin{aligned} \varPhi _N & = 77.5{^\circ }N + \varDelta , \\ \varPhi _0 & = 60.0{^\circ }N + \varDelta , \\ \varPhi_S &= 40.0{^\circ }N + \varDelta , \\ \varDelta &= -5.0{^\circ }, -2.5{^\circ }, 0{^\circ }, 2.5{^\circ }, 5.0{^\circ }, \end{aligned}$$

where for a particular day *Z* is Z500 at a given latitude ($\varPhi$) and longitude ($\lambda$), and $\overline{Z}$ is the climatological mean of Z500 for that particular day. For a more detailed explanation of the blocking detection algorithm see Barriopedro et al. (2006).

**Fig. 1:**
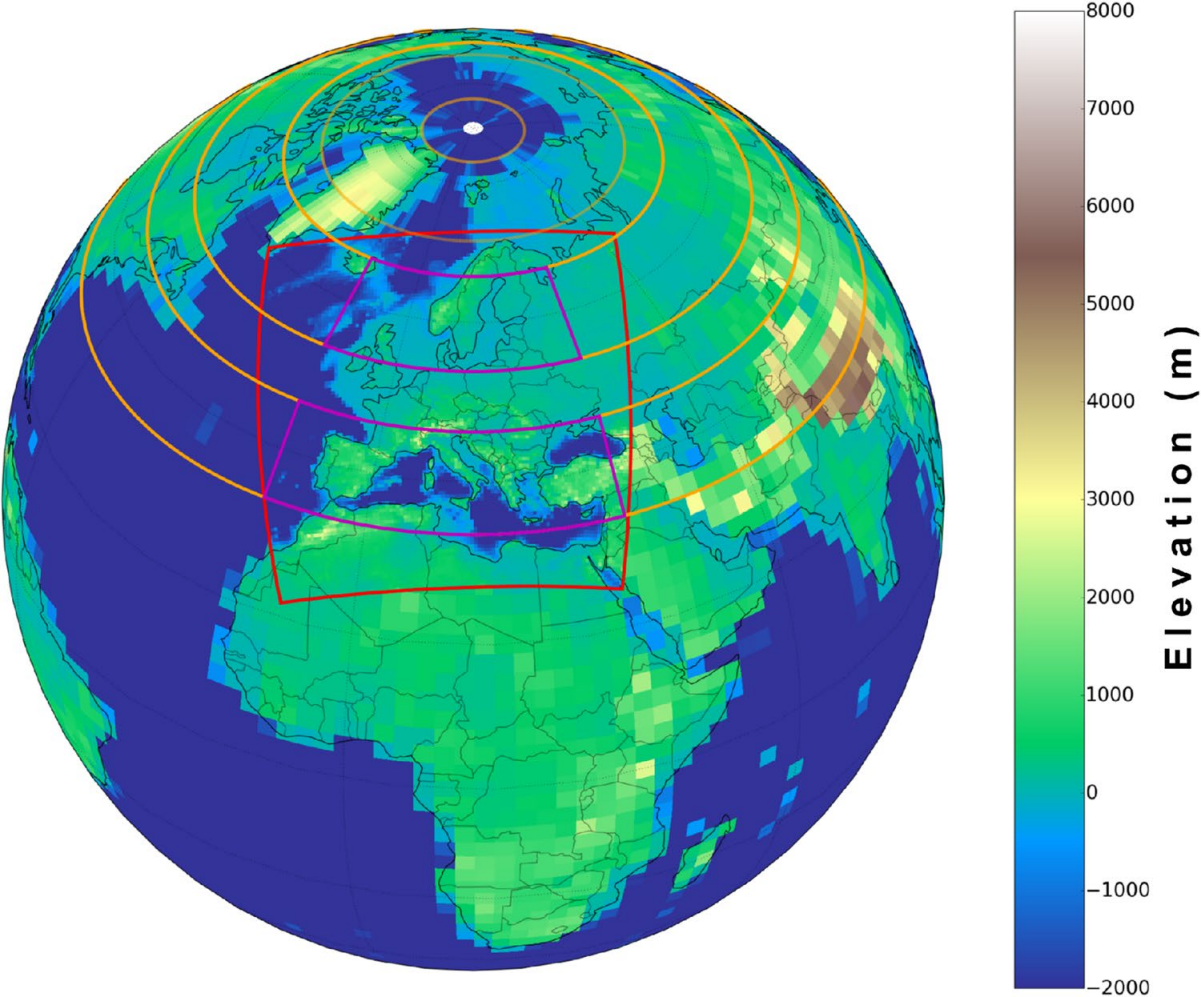
The EURO-CORDEX domain (red square). Orange lines depict the latitude bands (centered at $\varPhi _N, \varPhi _0$ and $\varPhi _S$) defined in the original blocking algorithm (Eqs. 1–3). The magenta domains depict the areas for which Z500 data of the RCMs have been used for the blocking detection scheme

In order to adapt the blocking algorithm, which requires Z500 data for the entire northern hemisphere, to the EURO-CORDEX RCM domain (see Fig. 1), we used RCM Z500 data over the region of [16.25$^\circ$W, 38.75$^\circ$E] and [33.75$^\circ$N, 66.25$^\circ$N] and ERA-Interim Z500 data for the remaining northern hemisphere. Further, we omitted the northward blocking criterion in the blocking detection (Eq. 2) to ensure that Z500 data was processed only intra-dataset wise. This simplification led only to marginal changes in the detected blockings (in the order of 1% of all days), since Eq. (2) is just set to guarantee the blocking detection and to exclude some few synoptic cases that are not blocking systems.

For every daily occurrence of the so-detected blocking events the detection scheme finds the grid point of maximum Z500 within the anticyclonic flow (see Barriopedro et al. 2006), called the blocking center (BC). Previous studies have shown that European blocking impacts on TAS and PR are different depending on the specific blocking location (cf. Sousa et al. 2017b, a). Thus, to obtain meaningful representations of the impact of blockings on surface parameters, we used the BC to derive time series of blocking days over three different sectors of the Euro-Atlantic region: the Eastern Atlantic (ATL, 30$^\circ$W–0$^\circ$E), Europe (EUR, 0$^\circ$E–30$^\circ$E) and Russia (RUS, 30$^\circ$E–60$^\circ$E). The rest of the days are cataloged as non-blocking days.

### Blocking bias decomposition

To evaluate a given RCM we decomposed the model bias in blocking and non-blocking components. For a given parameter (e.g. TAS and PR), the bias is defined as the difference between the climatological mean simulated parameter *X* and the corresponding observation *O*, $X-O$.

If $X_B$ ($O_B$) and $X_N$ ($O_N$) represent the mean conditions in the model (reanalysis) during blocking and non-blocking days, respectively, then the climatological mean parameter in the model and in observations can be decomposed as follows:

4 $$\begin{aligned} X & = f_X \cdot X_B + (1-f_X) \cdot X_N , \end{aligned}$$

5 $$\begin{aligned} O & = f_O \cdot O_B + (1-f_O) \cdot O_N , \end{aligned}$$

where $f_X$ ($f_O$) is the frequency of blocking days, and $1-f_X$ ($1-f_O$) is the frequency of non-blocking days in the model (reanalysis).

In our case, $f_O$ has been derived from the ERA-Interim reanalysis data, and *O* from the VALUE ECA 86 v2 dataset. We further perform an attribution of falsely and truly detected blocking and non-blocking days. With such an approach, blocking days detected in both, ERA-Interim and the RCM, are considered correctly detected blocks [true positive (*TP*)], while simultaneously detected non-blocking days in both datasets correspond to true negative (*TN*). From the point of view of the reanalysis, blocking days in ERA-Interim that are not captured by the RCM represent false negative (*FN*) detections, while non-blocking days in ERA-Interim that are detected as blocking days in the RCM are false positive (*FP*) detections. Accordingly, the cross-comparison of ERA-Interim and the RCM output allows the following decomposition of days, as shown in Table 3 and Eqs. (6) and (7):

**Table 3:** Classification of TN, TP, FN and FP terms according to blocking and non-blocking frequencies of observation and model

	$1 - f_X$	$f_X$
$1 - f_O$	TN	FP
$f_O$	FN	TP

6 $$\begin{aligned} f_X & = FP + TP \text { and } 1 - f_X = FN + TN , \end{aligned}$$

7 $$\begin{aligned} f_O & = FN + TP \text { and } 1 - f_O = FP + TN . \end{aligned}$$

Using this partitioning, the bias of a model ($X-O$) can be rearranged as follows:

8 $$\begin{aligned} X-O = FP \cdot (X_B - O_N) + FN \cdot (X_N - O_B) + TP \cdot (X_B - O_B) + TN \cdot (X_N - O_N), \end{aligned}$$

where the first two terms represent the contribution from a bias in blocking frequency (BF), due to either *FP* or *FN* detections, and the last two parts are the contribution from the biases in blocking and non-blocking patterns, respectively.

## Results

### Biases in blockings

**Fig. 2:**
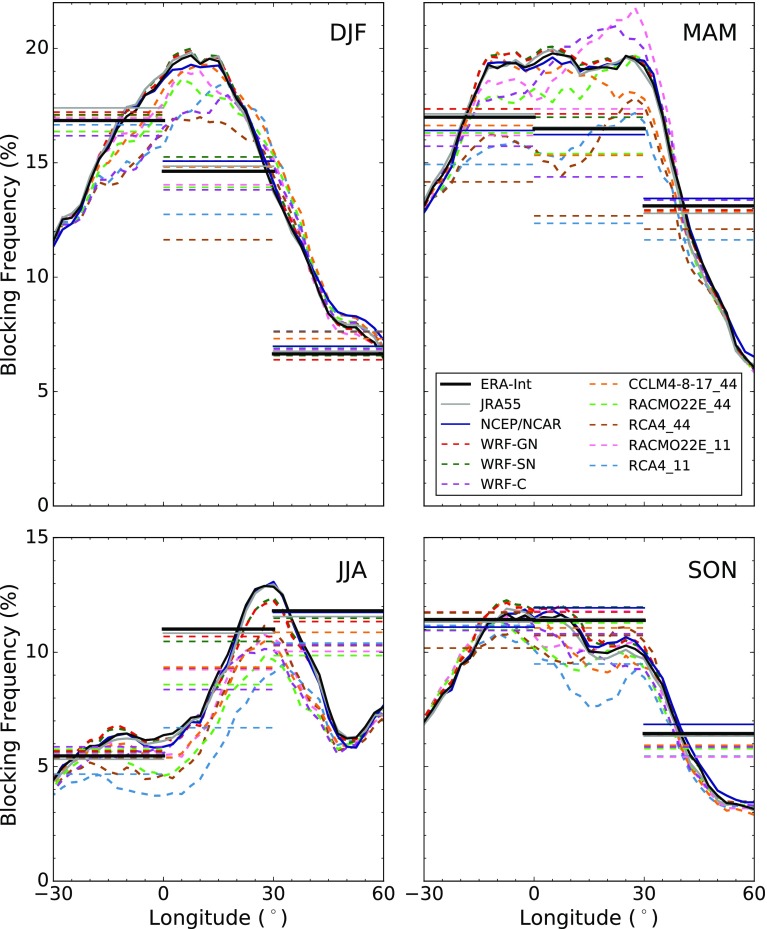
Seasonal mean frequency of blocked longitudes (expressed in percentage of all days within the respective season) over the Euro-Atlantic region for different reanalyses (solid lines) and RCMs (dashed lines). The frequency of BCs for the three different sectors (ATL, EUR and RUS) is indicated by the horizontal lines

The blocking detection scheme was applied to the three reanalysis products and the eight different RCMs. Figure 2 shows the longitudinal distribution of seasonal mean BF expressed in percentage of days. The results are in good agreement with well-known blocking distributions cited in literature, showing the distinct winter peak over the eastern Atlantic and the summer peak located further east over continental Europe (e.g. Barriopedro et al. 2006).

All reanalysis products show a high level of agreement. The two nudged RCMs (WRF-GN and WRF-SN) only display small deviations from the driving reanalysis. However, the free-running RCMs from EURO-CORDEX generally under-represent the blocking days throughout the year, especially in summer, when the simulated BFs can drop to almost half of those in the ERA-Interim reanalysis. There are only small over-estimations for WRF-C and RACMO22E_11 in spring. The horizontal lines in Fig. 2 indicate the relative frequency of the BCs being located in the three different sectors ATL, EUR and RUS. The model under-representation of blocking in terms of the BC is visible in the different sectors, particularly in EUR. This BC bias is of the same order as that in the longitudinal BF, indicating that the blocking underestimation in longitude is attributable to a lower BF rather than to a smaller blocking extension.

**Fig. 3:**
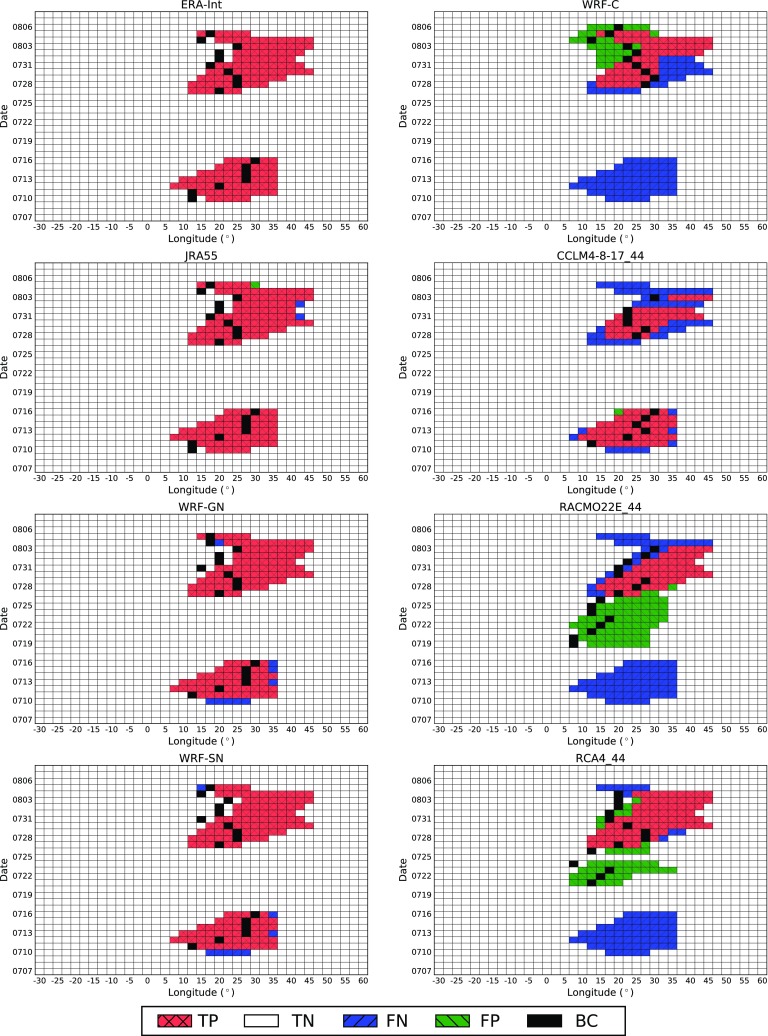
Hovmöller diagram of blocked longitudes between 4 July and 8 August 1994 for two reanalyses and different RCMs. Red squares indicate blocked longitudes (TP) and white squares non-blocked longitudes (TN) detected in ERA-Interim and the given dataset. Green squares depict blocked longitudes detected in the considered dataset but not in ERA-Interim (FP). Blue squares show blocked longitudes detected in ERA-Interim but not in the given dataset (FN). Black squares indicate the BC detected in each dataset

Figure 3 shows the specific blocking situation during the severe European heatwave of 1994 (Russo et al. 2015). A blocking event of 7 days centered around 20$^\circ$E was followed 10 days later by a second episode of 10 days at the same location. There is a good agreement between ERA-Interim, JRA55 (two panels on the top left) and NCEP/NCAR (not shown). The blocking events detected by the two nudged WRF RCMs (WRF-GN and WRF-SN, two panels on the bottom left) are also in good agreement with those of ERA-Interim, while the freely run EURO-CORDEX RCMs (right column) show more deficiencies in reproducing the correct blocking pattern in respect to both, spatial characteristics and temporal features.

**Fig. 4:**
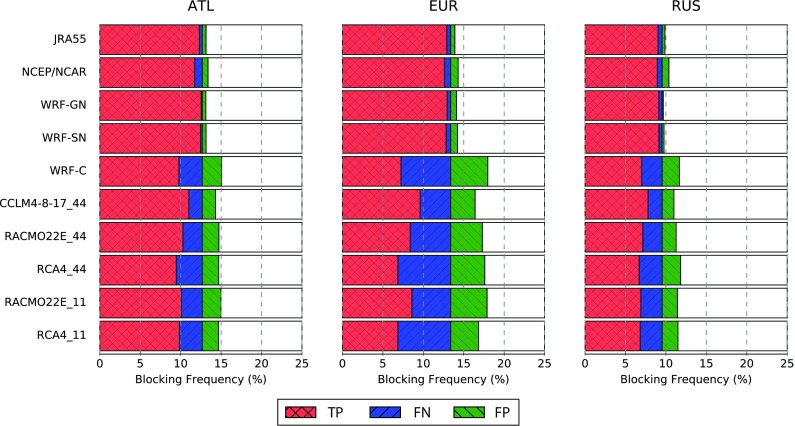
Relative annual BC frequencies in reanalyses and RCMs over the Eastern Atlantic (ATL), European (EUR) and Russian (RUS) sector. Frequencies are expressed in percentage of all annual days with respect to ERA-Interim (*TP* true positive, *FP* false positive, *FN* false negative)

**Fig. 5:**
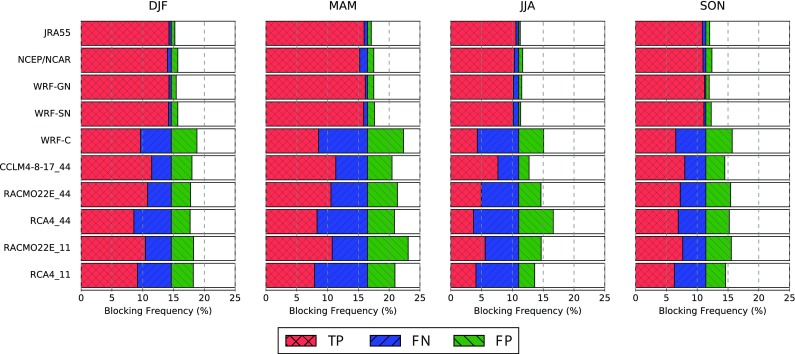
As Fig. 4 but for the European sector in winter (DJF), spring (MAM), summer (JJA) and autumn (SON)

The underestimated BFs in the RCMs are also visible in the relative frequencies of BCs presented in Fig. 4, which have been partitioned into TP, FN and FP according to Table 3 (the remaining fraction of days correspond to TN detections). The nudged RCMs indicate a small misrepresentation of blocking days (i.e., falsely positive or negative detections) that is even slightly lower (from 0.1 to 0.8% of all days) than that of the reanalyses JRA55 and NCEP/NCAR (from 0.3 to 0.9%), with no clear differences between the spectral and the gridded approach. Nevertheless, the fraction of FN and FP blocks for the remaining RCMs is higher, lying between 1.5 and 6.5%. With the exception of the nudged models, the total of false components (FP and FN) corresponding to blocking and non-blocking days detected only by the model, can amount to roughly the number of blocks detected simultaneously by ERA-Interim and the model (TP). All RCMs show the largest deviations over the EUR sector, which is located in the center of the RCM domain, where the RCMs’ own dynamics act the most. Moreover, there are no clear improvements seen in EURO-CORDEX RCMs with higher resolution. A seasonal analysis indicates that the largest absolute deviations are generally found in spring, while the largest deviations relative to the total number of blockings occur in summer (see Figs. 5, S1, S2 in the Supplementary Material).

### Biases in the representation of surface anomalies

As we have shown, the nudged RCMs perform better than the EURO-CORDEX RCMs, which, in turn, do not display large differences among them. Thus, in the remaining of this paper, and for simplicity, we will only show the results for the nudged RCMs as well as for the WRF RCM in climatic mode (WRF-C) as representative of the EURO-CORDEX RCMs.

**Fig. 6:**
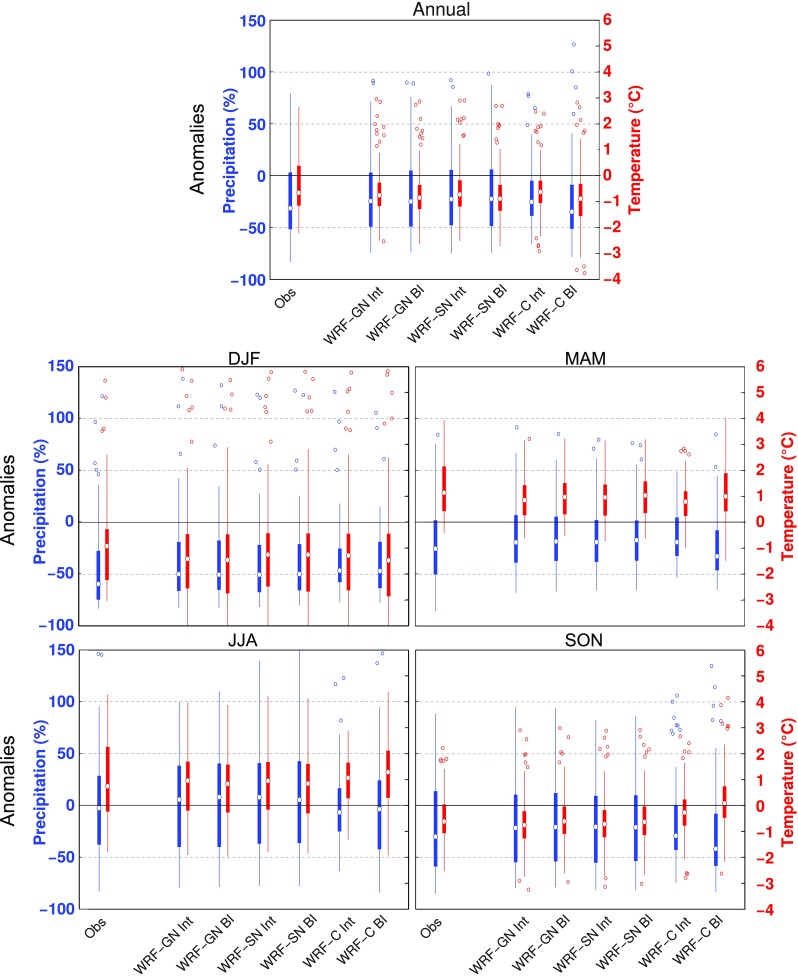
Boxplots indicating PR (in percentage of normals, blue) and TAS (in $^\circ$C, red) anomalies for EUR blocking days in the observations and three RCMs (WRF-GN, WRF-SN and WRF-C). Anomalies are obtained by using the blocking index of the model (BI) and ERA-Interim (Int). The boxes indicate the first and third quartiles, the whiskers extend to a maximum of 1.5 times the interquartile range, and flyers show data larger and smaller than the whiskers. Note that the boxplots represent the spatial distribution of the anomalies (i.e. the anomalies at the 86 station locations)

Figure 6 shows boxplots of the annual and seasonal TAS (in red) and PR (in blue) anomalies during blocking days over EUR for the observations, the nudged WRF runs and WRF-C. Anomalies have been calculated with respect to the climatological annual cycle of the full period of the respective dataset and were derived for the 86 station locations and their nearest RCM grid points. These anomalies have been obtained by using the Z500 field (and hence the blocking days) of the given model (BI hereafter). To better understand the origin of the RCMs’ discrepancies in blocking-related anomalies we have additionally replaced the Z500 field of the RCM by that of ERA-Interim before obtaining the surface anomalies of blocking for each RCM (this approach is referred to as Int, hereafter). From the point of view of the models, the difference between BI and Int is that the former includes non-blocking days in ERA-Interim detected as blocking by the RCM (FP), while the later includes blocking days in ERA-Interim not captured by the RCM (FN).

On the annual scale (top panel of Fig. 6), blocking situations are associated with cooling and reduced precipitation, with opposite but much weaker anomalies occurring during non-blocking days (not shown). All RCMs perform well in terms of the spatial distribution of TAS and PR anomalies. In particular, the seasonally contrasting behavior, with blocking inducing cooling in the cold seasons (DJF and SON) and warming in the warm seasons (MAM and JJA), is reasonably captured by the RCMs, although the free running WRF-C model indicates some deviations from the observed median temperatures during autumn. Different to TAS, the PR reductions associated to blocking are observed through most of the year, being larger in winter, and they are reproduced by all RCMs, albeit with a reduced spread in WRF-C.

Overall there are small differences in the blocking-related anomalies between the BI and Int groups. As the nudged WRF runs are strongly tied to the driving data, they show small FP and FN terms, and the blocking-related anomalies of TAS and PR are almost indistinguishable between BI and Int approaches. Imposing the ERA-Interim blocking days in the WRF-C model reduces most biases in TAS (for the annual mean and in DJF, JJA and SON) and some biases in PR (for SON), with similar results for the two other sectors (see Figs.  S3, S4 in the Supplementary Material) and the remaining EURO-CORDEX RCMs (not shown).

**Fig. 7:**
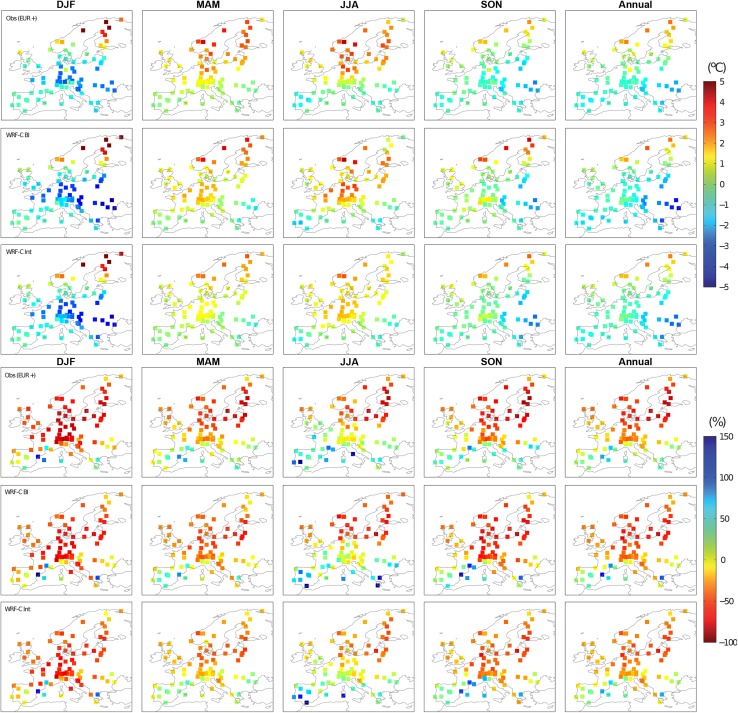
Seasonal and annual TAS (in $^\circ$C, row 1-3) and PR (in percentage of normals, row 4–6) anomalies during blocking days over the EUR sector. Rows 1 and 4 show the observed anomalies using the the blocking index calculated from ERA-Interim. Rows 2 and 5, and rows 3 and 6 show the anomalies in the WRF-C RCM using the blocking index calculated from the RCM (WRF-C BI) and the blocking index from the ERA-Interim (WRF-C Int), respectively

The observed spatial distributions of the blocking-related TAS and PR anomalies are characterized by warmer temperatures in Scandinavia and colder temperatures in southern and central Europe, as well as by overall dryer conditions (Fig. 7, last column). WRF-C reproduces these patterns reasonably well (see Fig. 7; WRF-C BI, rows 2 and 5; WRF-C Int, rows 3 and 6). For annual means, applying the ERA-Interim blocking days usually yields a better spatial agreement with TAS observations than using the blocking index defined by the model, while the opposite is the case for PR (cf. Table 4 listing root mean square errors of the spatial fields of the two aggregations presented in Fig. 7). The largest improvements in the spatial representation of TAS anomalies are achieved in the cold seasons (DJF and SON) when using Int, while the same approach leads to some deteriorations of PR anomalies in the transitional seasons (MAM and SON). For the two other sectors, the model shows a similar behavior to that found for EUR, but is more invariant to the applied blocking index (BI or Int, see Figs.  S5, S6 in the Supplementary Material).

**Table 4:** Seasonal and annual root mean square errors of the spatial fields of TAS ($^\circ$C) and PR (percentage of normals) for the two different EUR blocking aggregations (BI and Int) in the WRF-C model, as presented in Fig. 7

	TAS ($^\circ$C)		PR (%)	
	BI	Int	BI	Int
ANNUAL	0.61	0.52	15.8	17.2
DJF	1.04	0.95	20.2	20.2
MAM	0.55	0.68	19.7	21.1
JJA	0.86	0.87	36.1	35.6
SON	0.96	0.61	25.4	27.0

See text for details

Depending on the season, these results indicate some small improvements in the representation of surface fields after correcting the RCM biases in blocking days in the case of TAS and some deteriorations in the case of PR. However, general statements are challenging. The different responses of TAS and PR to the RCM correction may be due to varying influences of FP (affecting BI) and FN (affecting Int) days in the overall biases. This question will be further addressed in the next section.

### Contributions of blocking to biases in the surface fields

**Fig. 8:**
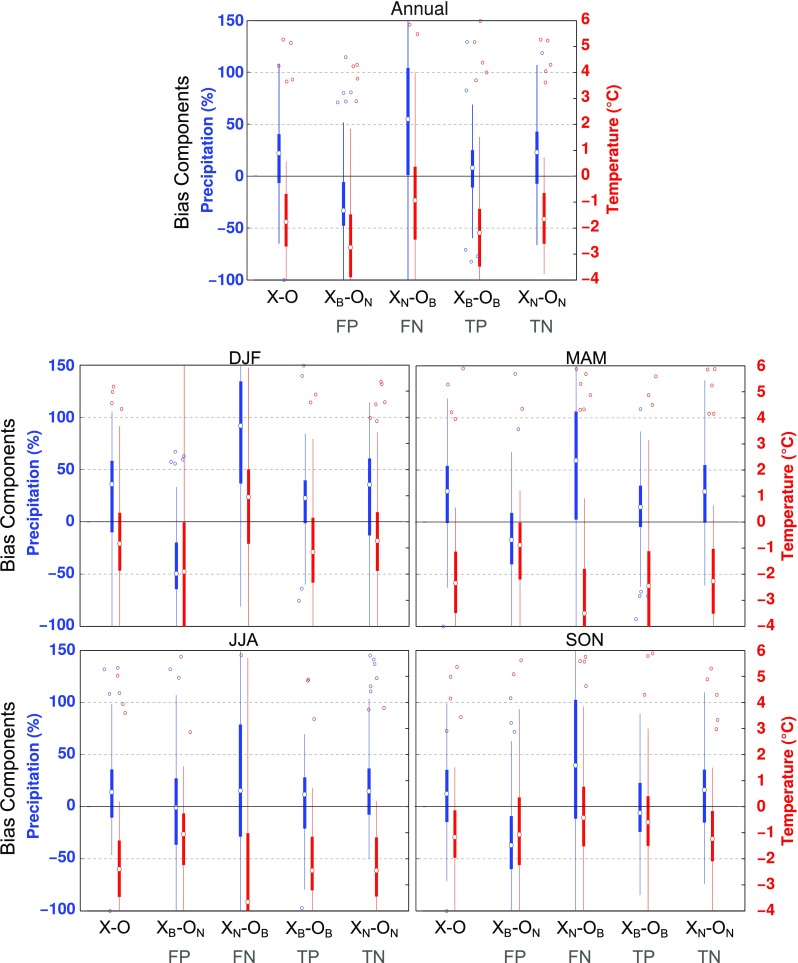
Boxplots showing single bias components of WRF-C (Eq. 8) for PR (in percent, blue) and TAS (in $^\circ$C, red) for EUR blocking. The bias components of PR have been calculated with respect to the observed climatological values (e.g. $(X_N - O_B)/O$). The boxes indicate the first and third quartiles, the whiskers extend to a maximum of 1.5 times the interquartile range, and flyers show data larger and smaller than the whiskers. Note that the boxplots represent the spatial distribution of the bias with respect to observations (i.e. the bias at the 86 station locations)

This last section investigates to what extent BF biases and biases in blocking-related surface patterns contribute to the overall bias of RCMs using Eq. (8). BF biases are related to FP and FN terms in Eq. (8), whereas biases in blocking and non-blocking patterns are given by the TP and TN components, respectively. As the biases of the nudged simulations are small, we will focus on the WRF-C model only, which is representative of the EURO-CORDEX RCMs. The WRF-C RCM has been shown to exhibit a systematic cold and wet bias (Katragkou et al. 2015). Figure 8 shows the climatological biases in TAS and PR (i.e., $X-O$) for our station locations, as well as the corresponding mean biases during FP, FN, TP and TN days. At annual scales WRF-C has a negative TAS bias of about −  1.8$^\circ$C and a positive PR bias of 20% (median values in the top panel of Fig. 8). This bias is roughly of the same order during situations not associated to blocking (TN), as measured by $X_N-O_N$, which are much more frequent than situations connected to blocking (TP, FP and FN). Similar to the climatological biases, blocking situations detected in ERA-Interim and the model (TP) lead to wetter and colder conditions than in observations (see the term $X_B-O_B$ in Fig. 8). However, these days contribute differently to TAS and PR full biases, increasing the former and reducing the latter. The cross terms (FP and FN), i.e. $X_B-O_N$ and $X_N-O_B$ in Fig. 8, tend to concentrate the largest deviations from (and display opposite effects in) the climatological biases.

At seasonal scales the climatological model biases tend to show the same sign as the annual bias (bottom panels of Fig. 8). The largest (smallest) biases towards wet conditions occur in winter (summer), arguably related to the seasonal cycle in PR. The mean biases of the different terms in Eq. (8) suggest that blocking effects in PR (drier conditions; Fig. 7) tend to decrease the climatological bias (wetter conditions). Thus, PR biases during blocking ($X_B - O_B$) are somewhat beneficial because they reduce the overall model bias, with the exception of winter. Accordingly, the wettest biases occur during FN days, which correspond to blocking situations (i.e., drier conditions), that are not captured by the model. Consistent with the coherent PR response to blocking throughout the entire year, this distribution of the single bias terms is observed all year round (and at the annual scale). As a consequence, the overall under-representations of BF (i.e. a large frequency of FN days) increases the mean wet biases, especially in DJF and MAM. This is also visible in Fig. S9, which shows the net contribution of the single bias terms to the climatological bias after weighting their mean biases by their fractional frequency as indicated in Figs.  4 and 5.

Different to PR, the largest cold biases in TAS occur in the warm seasons (MAM and JJA), and the contribution of the different terms to the overall bias varies through the year. In particular, FP days display the coldest biases in winter (DJF), whereas FN days account for the coldest biases in the warm seasons (MAM and JJA). These seasonal changes are in agreement with those observed in the blocking impacts in TAS. Thus, in the cold seasons, when blocks induce cooling, the mean bias is larger during FP days (i.e., false cold blocking conditions in the RCM). In the warm seasons, blocking is associated to warm conditions, and FN days display the largest mean cold bias, as the model misses the blocking-related warming. Given that FN days are more frequent than FP days, the under-representation of blockings in WRF-C amplifies the model bias in the warm seasons, but reduces it in the cold seasons (see also Fig. S9).

In summary, pattern biases (TP and TN) influence the WRF-C model bias much more strongly than the biases in BF (FP and FN), mainly due to the high fraction of TN days and the compensating effect of opposite biases in the false components with respect to the mean bias (Fig. S9). However, the higher the under-representation of blockings in an RCM, the higher the fractional FN term becomes in relation to the FP term. If the RCM is capable of reproducing the general anomaly structure during blocking situations, the higher fractional FN term will inevitably drag the overall model bias in the opposite direction of the blocking-related anomalies, leading to a warm (cold) bias in cold (warm) seasons and wet biases all year round. If the RCM shows a systematic wet bias, as in the case of WRF-C, the blocking underestimation would act to increase the overall bias. However, if the RCM is too dry, it would actually decrease the overall bias. As for TAS, false detections would lead to seasonal changes in terms of the overall bias. If the RCM is too warm, a blocking underestimation would be beneficial in the warm seasons and detrimental in the cold seasons, while the opposite would occur if the RCM is too cold, as observed in WRF-C.

## Summary and discussion

State-of-the-art EURO-CORDEX RCMs show a different representation of blockings than their driving data (ERA-Interim) mainly in the center of the RCM domain, where the RCMs’ own dynamics are less constrained by the boundary conditions. Our results indicate a general underestimation and a misrepresentation of up to 13% of all days for some seasons, including relevant episodes like the European heatwave of 1994. Hence, overall there is a deviation in the representation of atmospheric blocking over the modelling domain. The resolution of the RCMs does not have an influence on our results, running RCMs at higher resolutions alone is not sufficient for improving the representation of atmospheric blocking over the EURO-CORDEX domain. A stronger dependence of the RCM on the driving reanalysis could reduce the blocking frequency bias to less than 2% according to the results obtained with the two nudged WRF simulations.

Despite the biases in blocking frequency, the EURO-CORDEX RCMs are able to reproduce the basic blocking-related TAS and PR anomalies. Deviations in the representation of the surface anomalies compared to the observations are smaller for RCMs that are more conditioned to the driving reanalysis, indicating some influence of false detections in the overall surface biases, with no clear differences between the spectral and grid nudging. As results for the two different nudging techniques did not differ, spectral nudging may be preferred, as it grants the RCM more freedom to develop regional scale features.

Overall, the surface biases during blocking situations detected by the RCM (WRF-C) and the driving reanalysis are not very different from the mean biases, which are characterized by wetter and colder conditions than in the observations. Thus, blocking does not seem to contribute more than non-blocking days to the mean biases. While the overall model biases are mainly determined by pattern biases during the more frequent non-blocking days, there are substantial contributions of blocking frequency biases (i.e. FP and FN days), which are of opposite sign with respect to the mean bias. If these components are balanced, they would result in a partial cancellation. Nevertheless, in the case of blocking under-representation, missed blocks exceed falsely detected blocks, dragging the model bias in the opposite direction of blocking-related anomalies. Thus, the resulting effect of a blocking underestimation in the representation of surface fields can be beneficial or detrimental, depending on whether the systematic RCM bias is of equal or opposite sign to that of blocking-related anomalies.

According to our conclusions, it may be advisable to strongly condition RCMs to their driving data. Since we conducted our analysis with reanalysis boundary data alone, it could be rewarding to transfer the applied framework to RCMs driven by GCM data. Further, using derived blocking indices from the respective driving data (e.g. GCMs) could be enough to evaluate high-resolution blocking impacts over the EURO-CORDEX domain, as our results were similar when blocks of the driving data were used to evaluate blocking effects in surface anomaly fields. However, we strongly recommend a thorough evaluation of the large-scale atmospheric circulation when selecting the driving GCMs for RCM studies.

## Electronic supplementary material

Below is the link to the electronic supplementary material.

Supplementary material 1 (pdf 19 KB)

Supplementary material 2 (pdf 19 KB)

Supplementary material 3 (pdf 1615 KB)

Supplementary material 4 (pdf 1097 KB)

Supplementary material 5 (pdf 2116 KB)

Supplementary material 6 (pdf 2113 KB)

Supplementary material 7 (pdf 529 KB)

Supplementary material 8 (pdf 987 KB)

Supplementary material 9 (pdf 7 KB)

Supplementary material 10 (pdf 14 KB)

Supplementary material 11 (pdf 13 KB)
