# Supplementary figures and images for: Blocking representation in the ERA-Interim driven EURO-CORDEX RCMs
Source: Clim Dyn. 2018 Jul 30;52(5):3291–306. doi: 10.1007/s00382-018-4335-8 (PMC6424152; doi:10.1007/s00382-018-4335-8)

DJF

MAM

JJA

SON

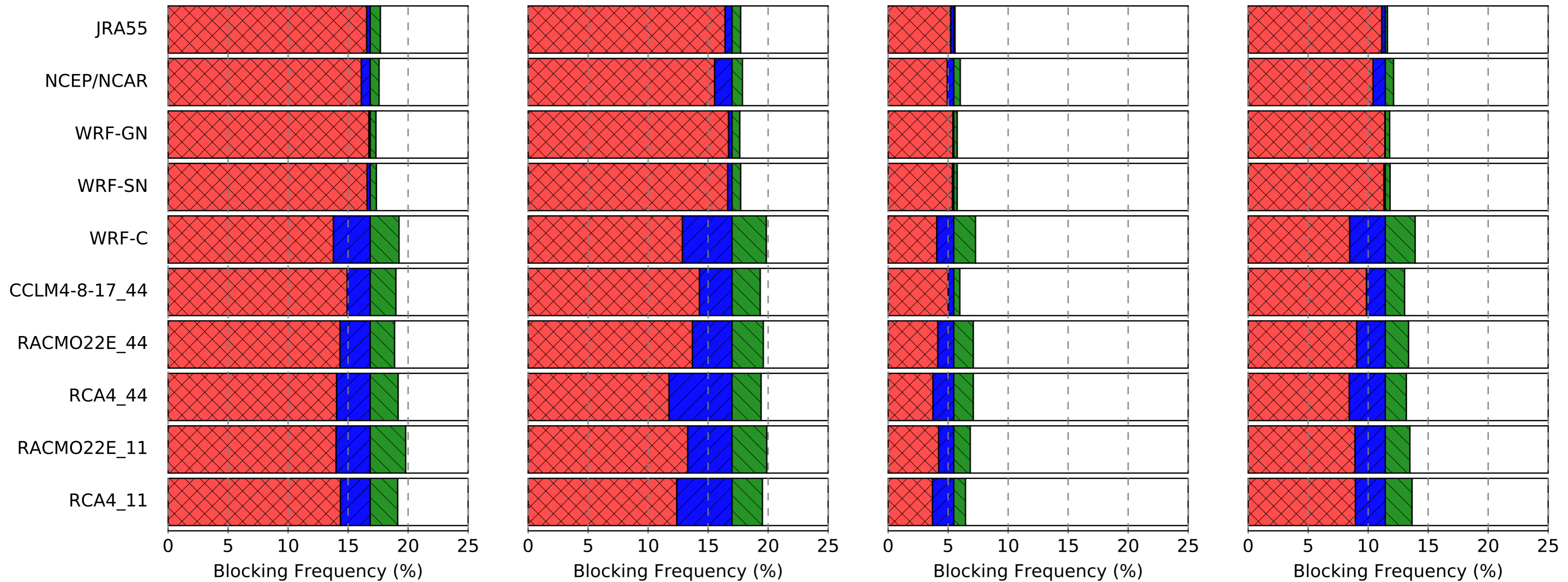

Supplement: Supplementary file 1 — Supplementary material 1 (pdf 19 KB) [file 382_2018_4335_MOESM1_ESM.pdf]

DJF

MAM

JJA

SON

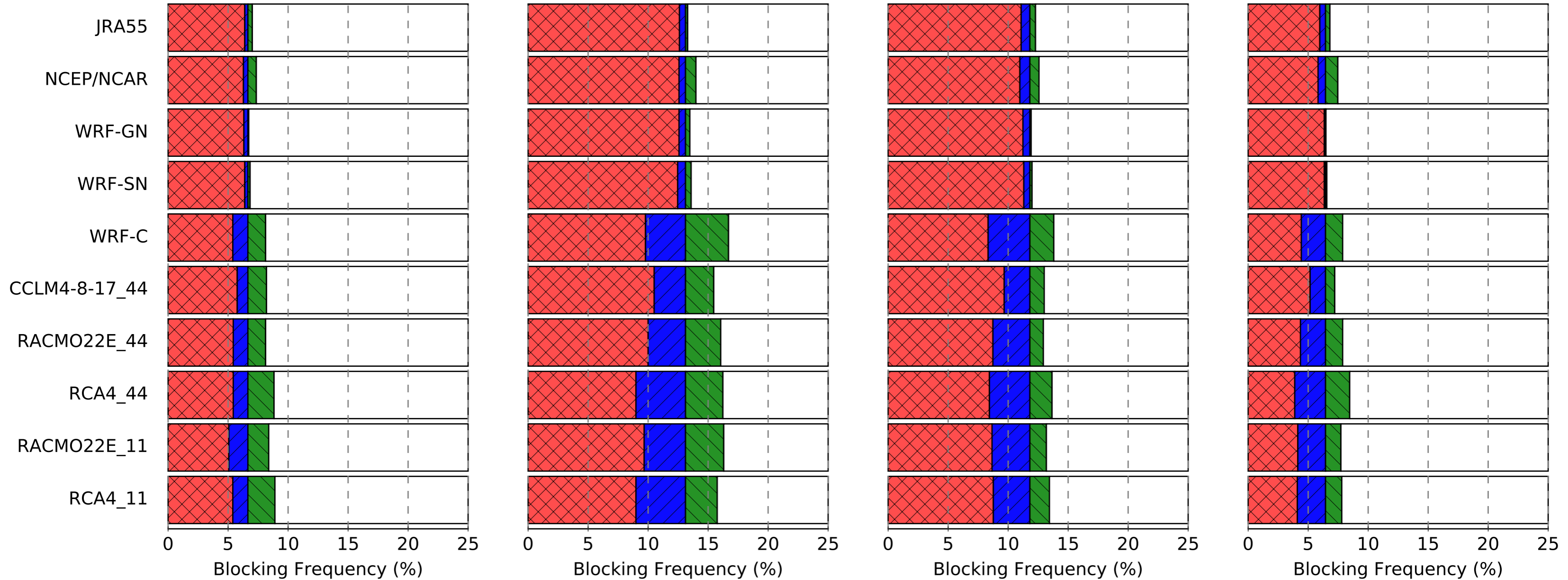

Supplement: Supplementary file 2 — Supplementary material 2 (pdf 19 KB) [file 382_2018_4335_MOESM2_ESM.pdf]

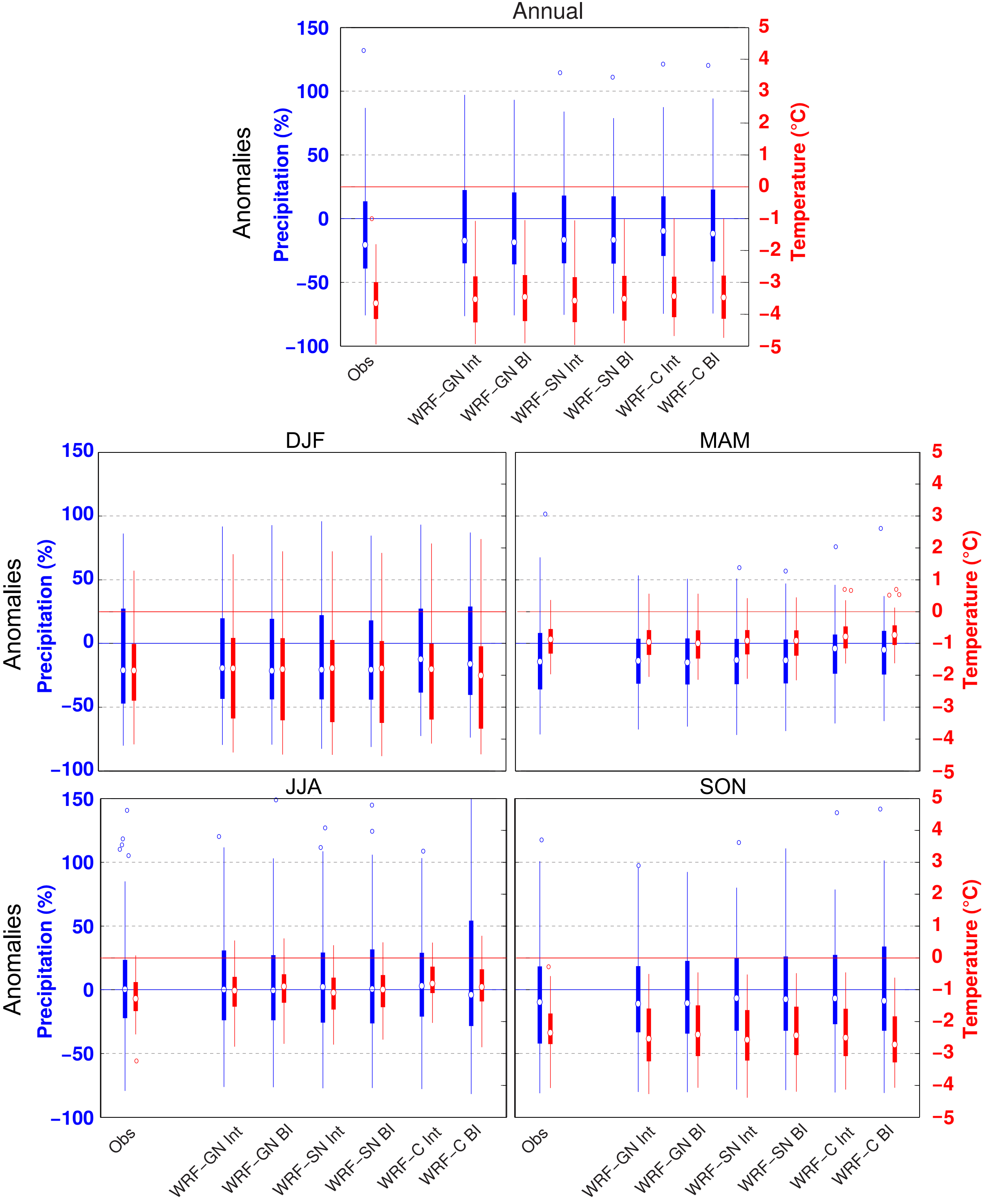

Supplement: Supplementary file 3 — Supplementary material 3 (pdf 1615 KB) [file 382_2018_4335_MOESM3_ESM.pdf]

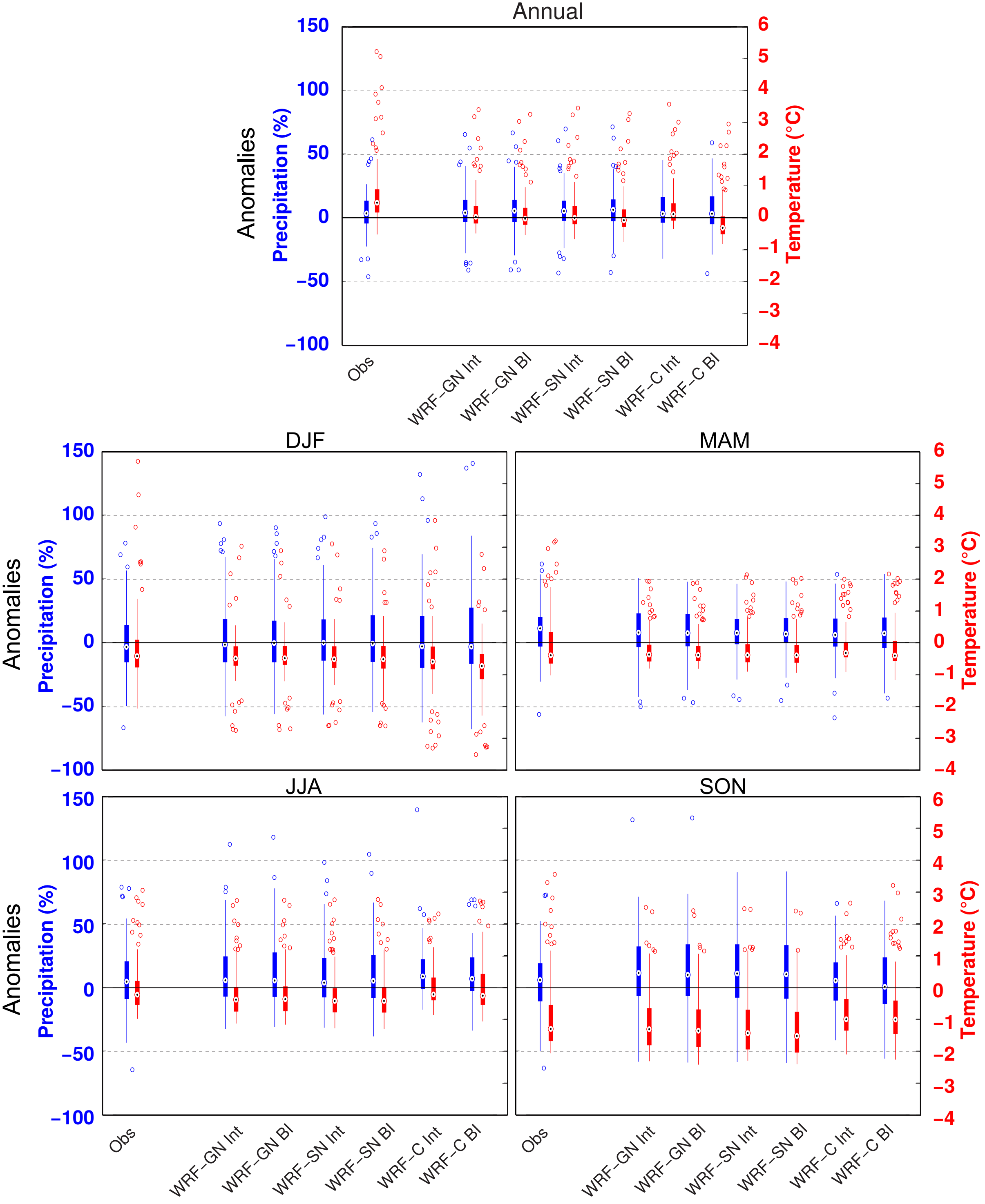

Supplement: Supplementary file 4 — Supplementary material 4 (pdf 1097 KB) [file 382_2018_4335_MOESM4_ESM.pdf]

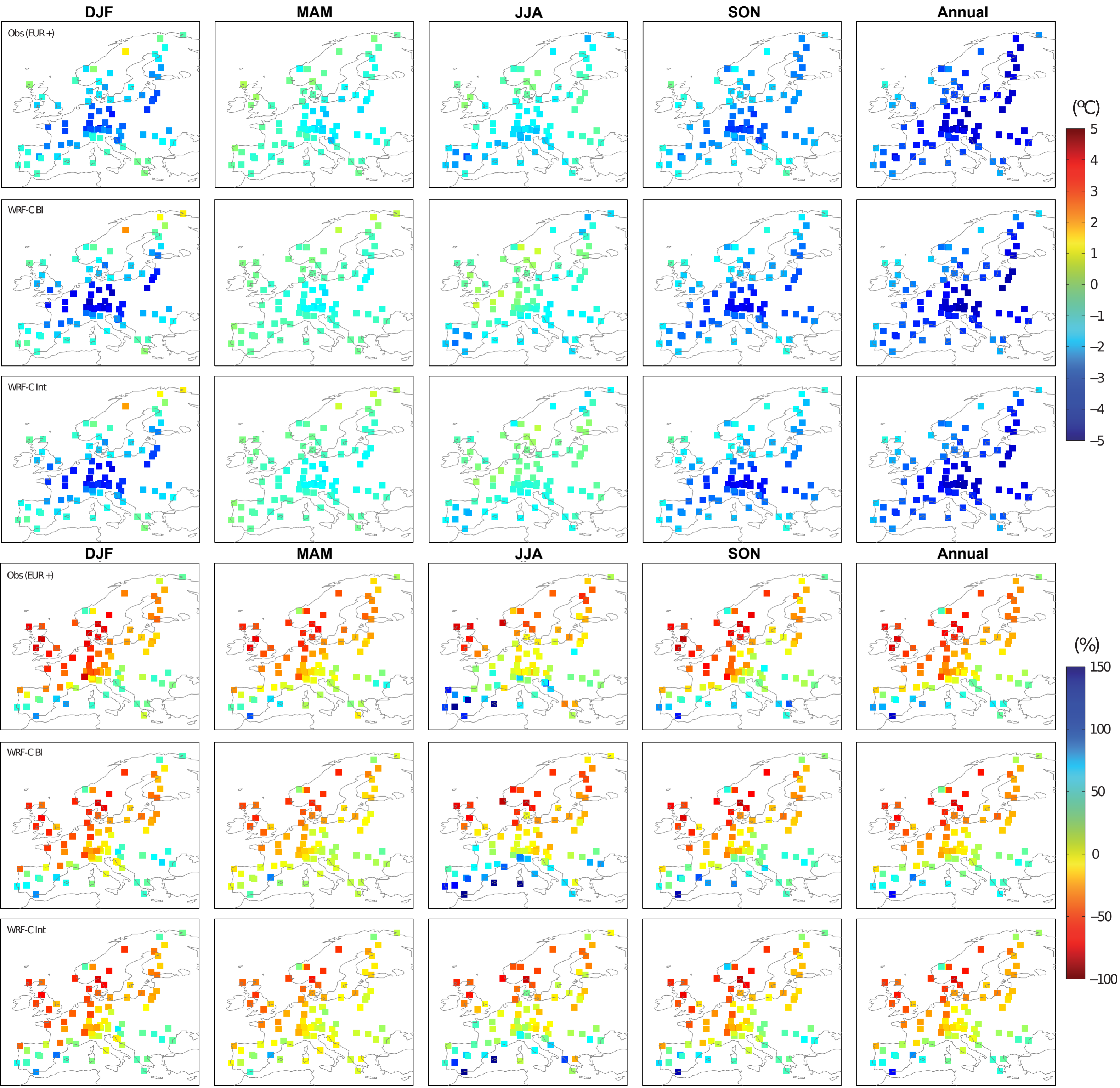

Supplement: Supplementary file 5 — Supplementary material 5 (pdf 2116 KB) [file 382_2018_4335_MOESM5_ESM.pdf]

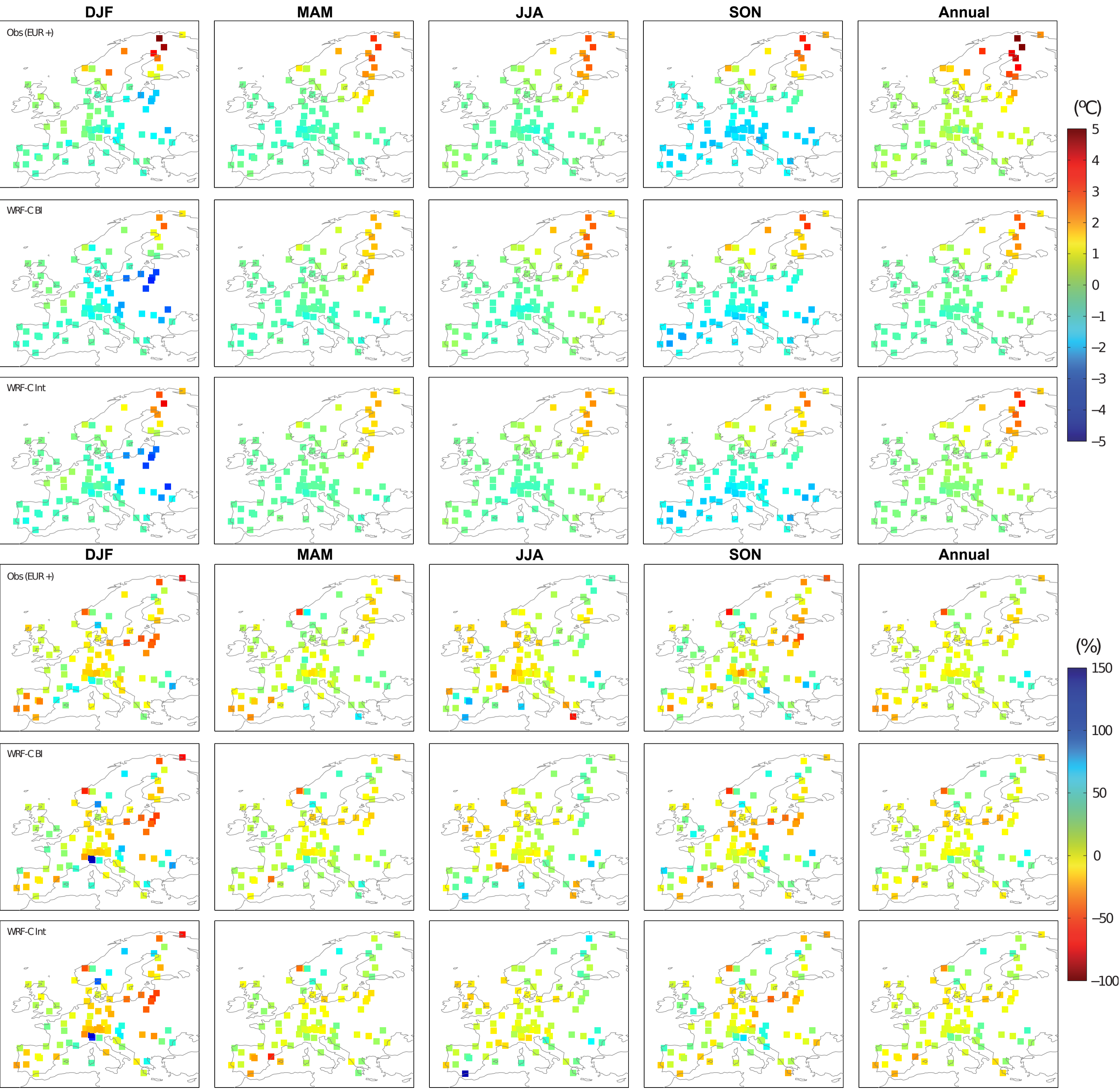

Supplement: Supplementary file 6 — Supplementary material 6 (pdf 2113 KB) [file 382_2018_4335_MOESM6_ESM.pdf]

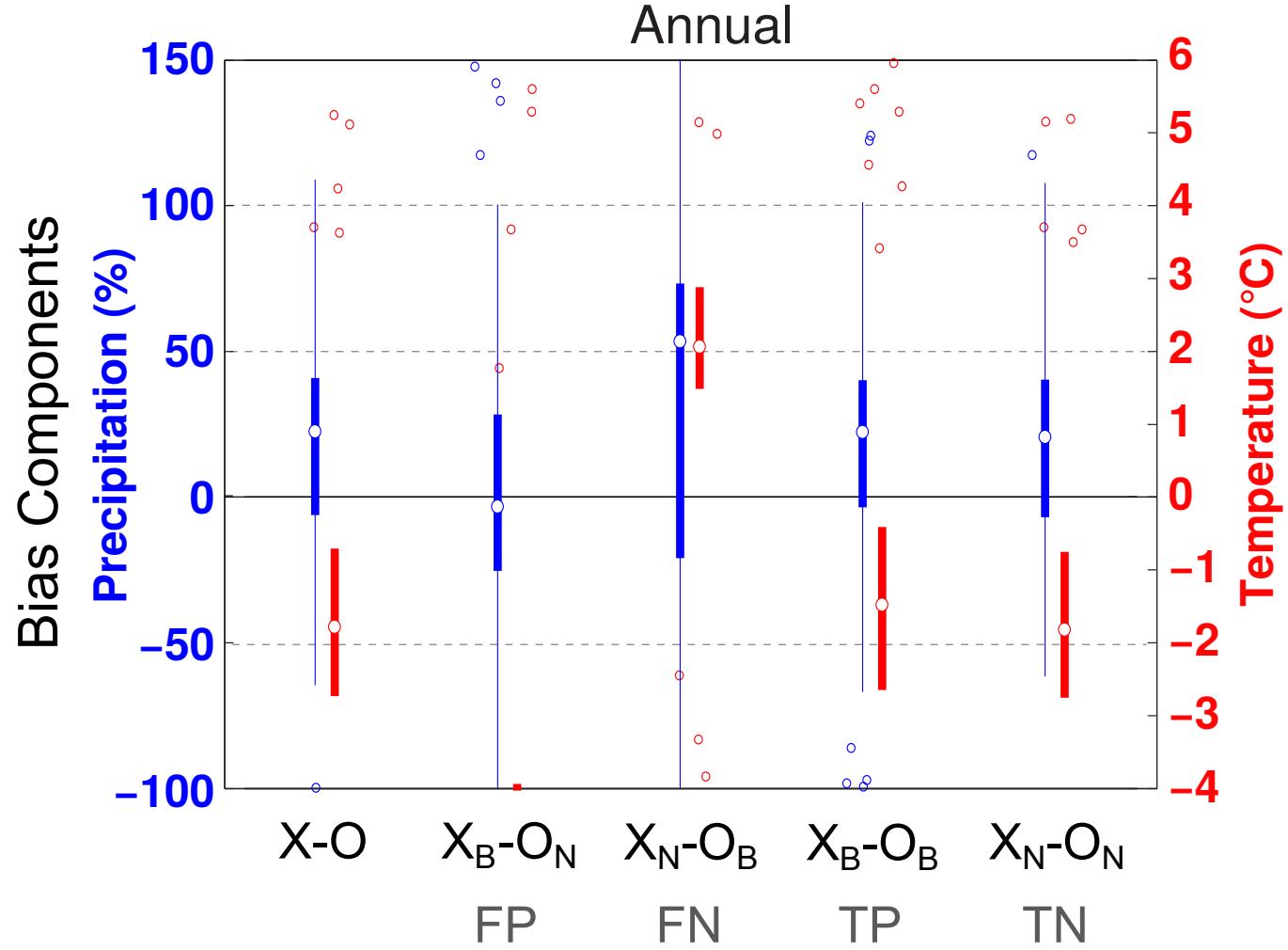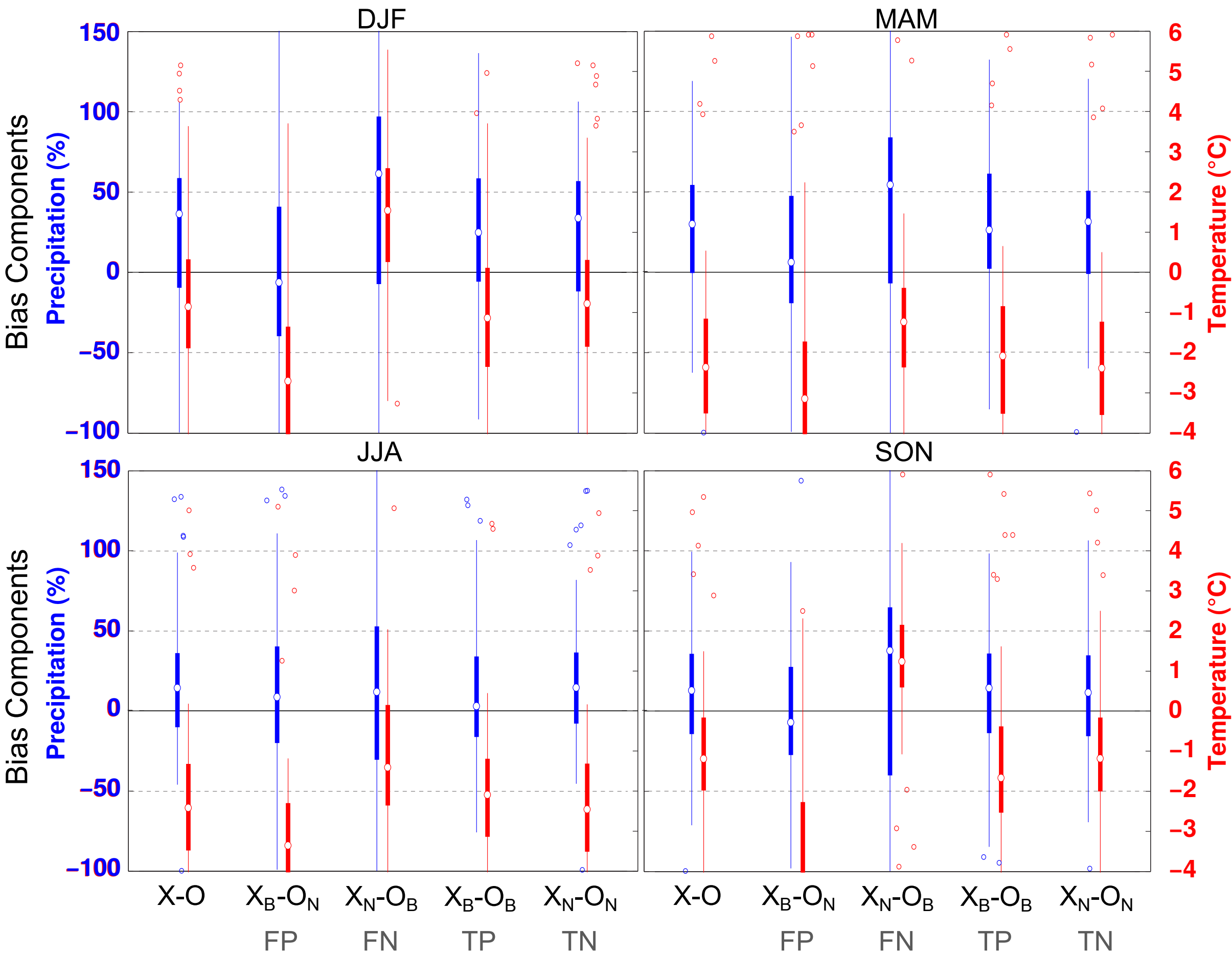

Supplement: Supplementary file 7 — Supplementary material 7 (pdf 529 KB) [file 382_2018_4335_MOESM7_ESM.pdf]

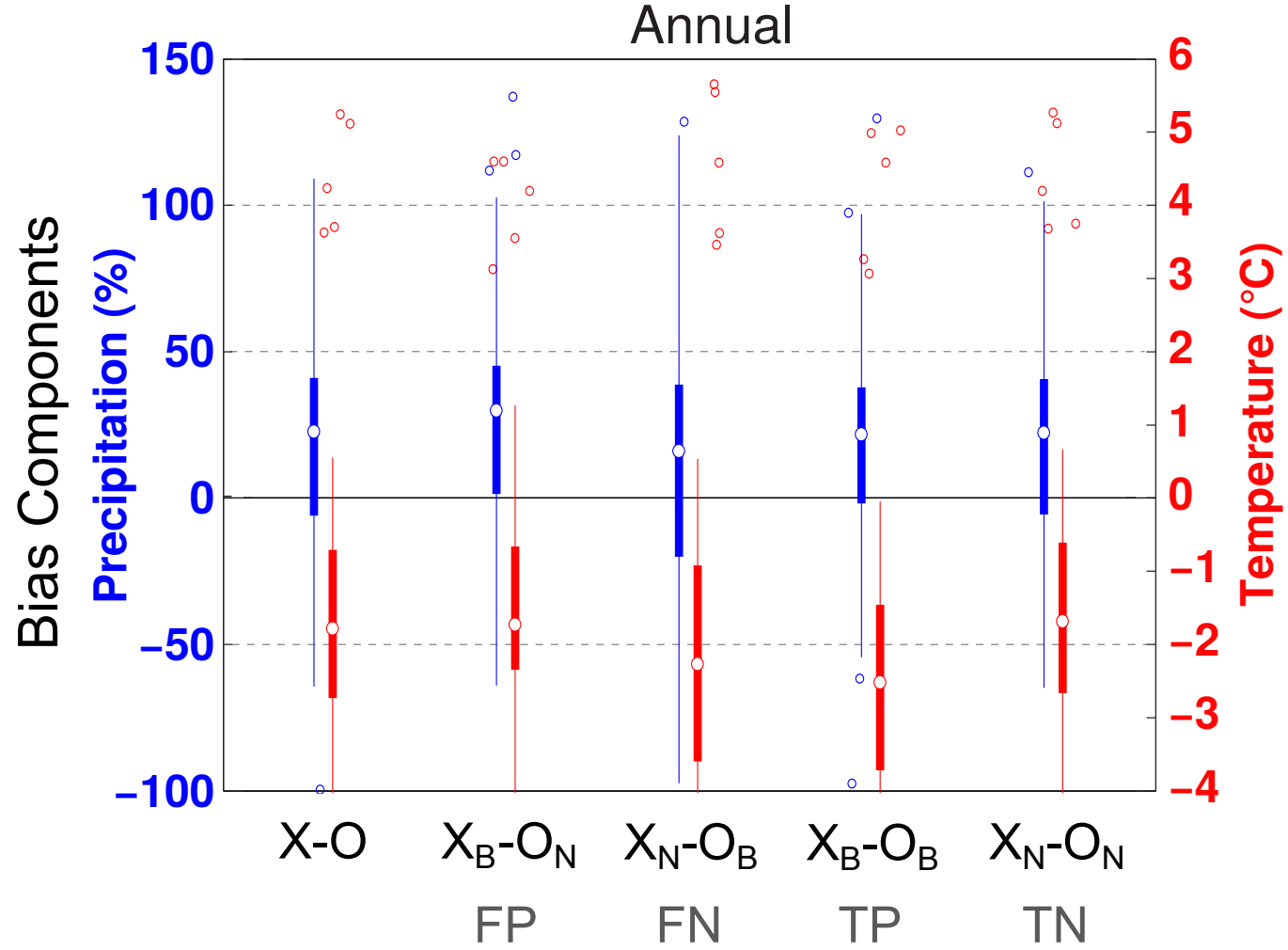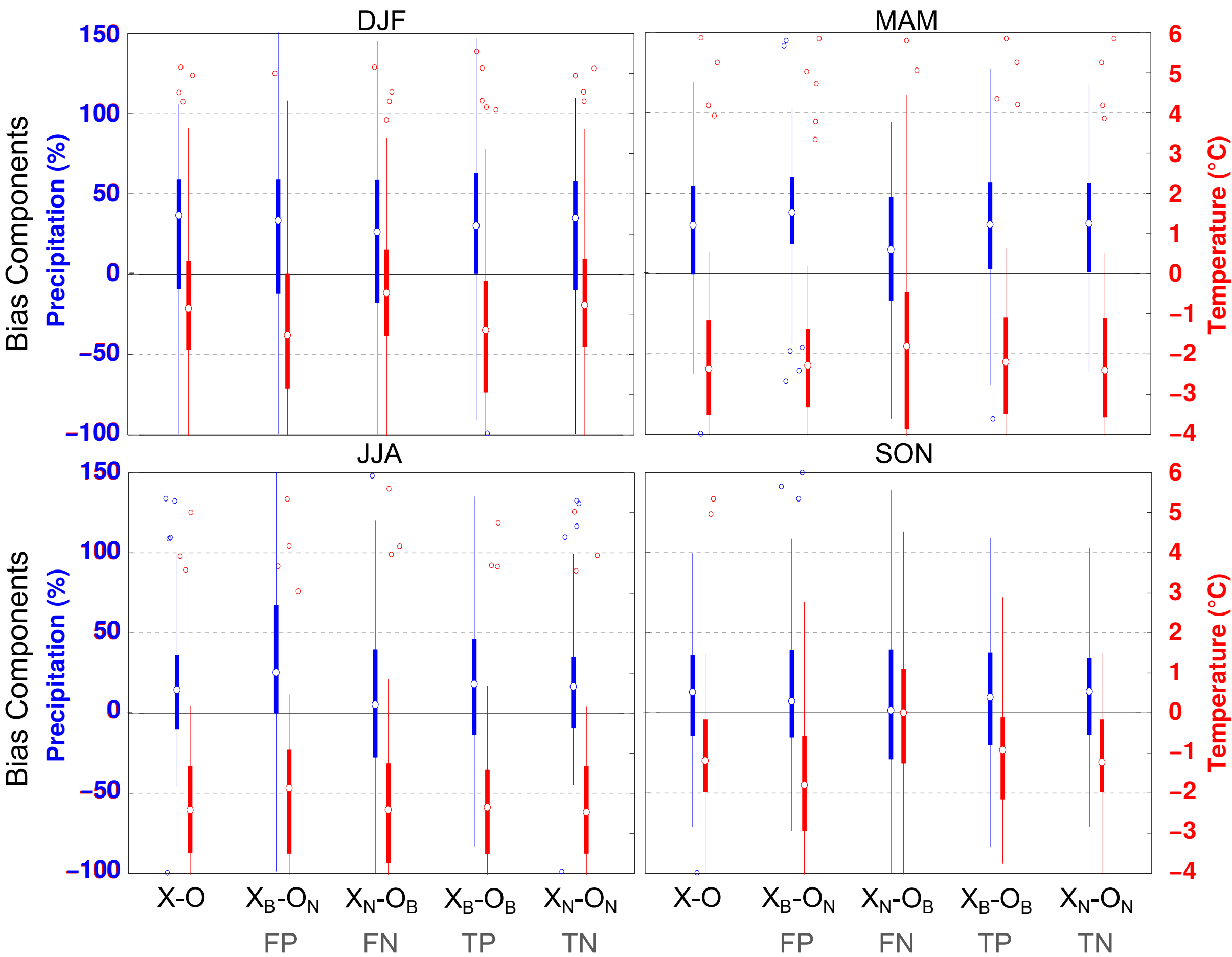

Supplement: Supplementary file 8 — Supplementary material 8 (pdf 987 KB) [file 382_2018_4335_MOESM8_ESM.pdf]

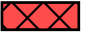

TP

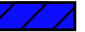

FN

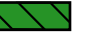

FP

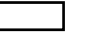

TN

Supplement: Supplementary file 9 — Supplementary material 9 (pdf 7 KB) [file 382_2018_4335_MOESM9_ESM.pdf]

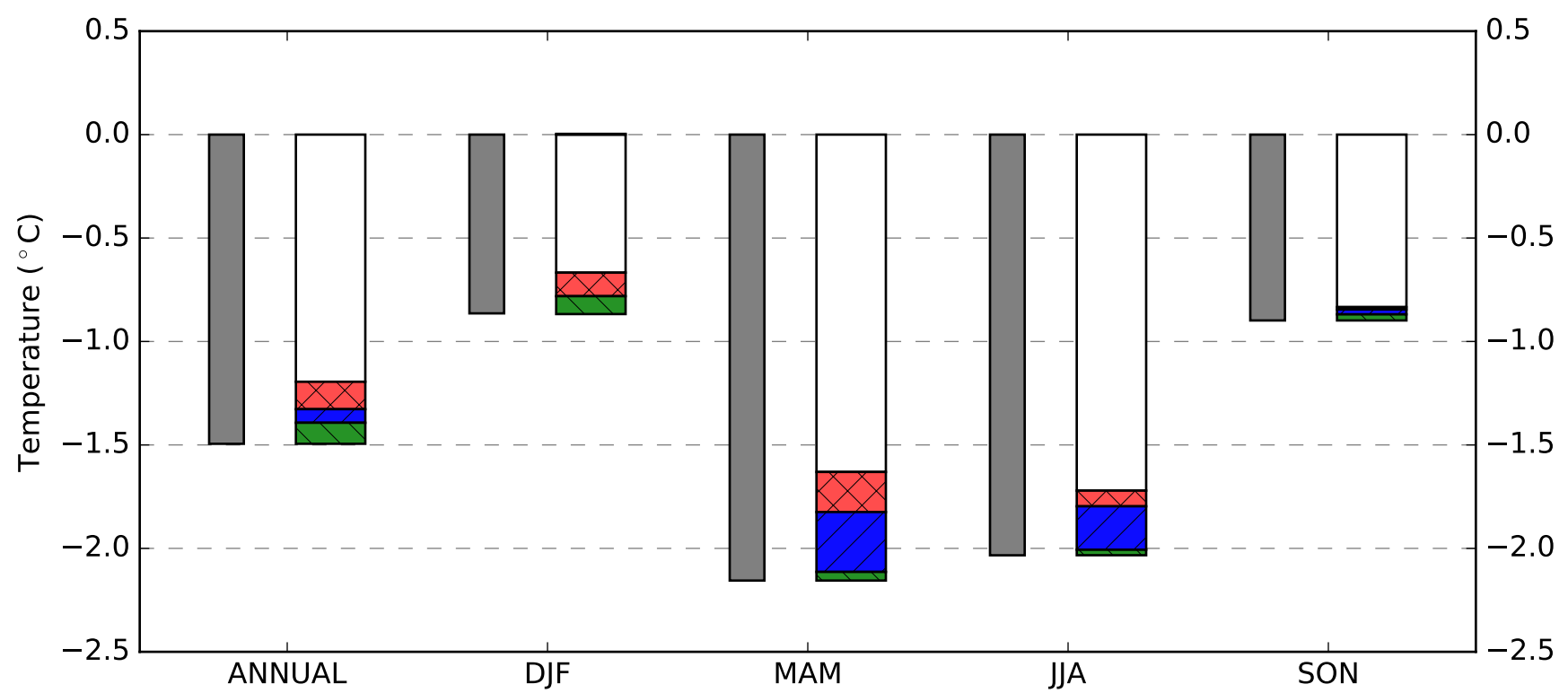

Supplement: Supplementary file 10 — Supplementary material 10 (pdf 14 KB) [file 382_2018_4335_MOESM10_ESM.pdf]

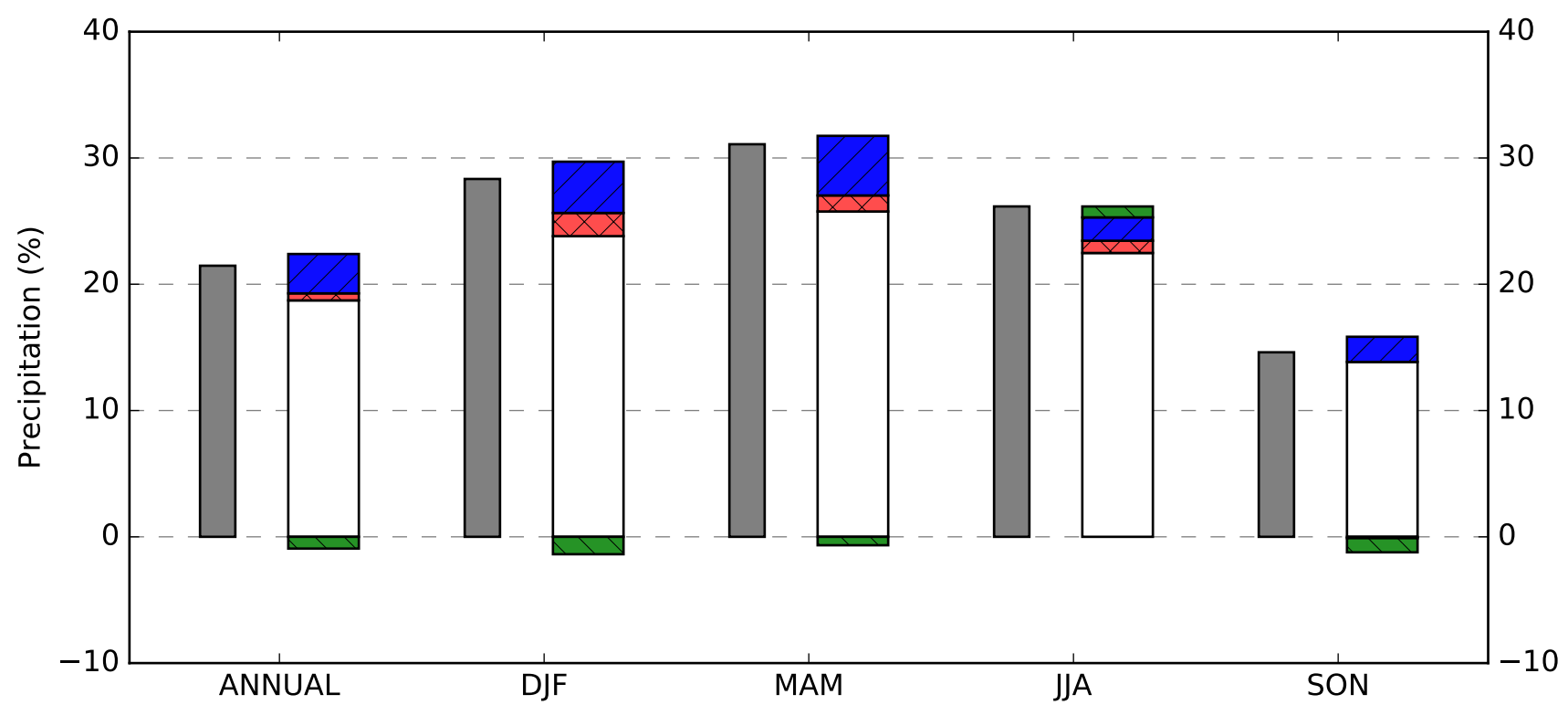

Supplement: Supplementary file 11 — Supplementary material 11 (pdf 13 KB) [file 382_2018_4335_MOESM11_ESM.pdf]
